# Influence of Magnetic Field with Schumann Resonance Frequencies on Photosynthetic Light Reactions in Wheat and Pea

**DOI:** 10.3390/cells10010149

**Published:** 2021-01-13

**Authors:** Vladimir Sukhov, Ekaterina Sukhova, Yulia Sinitsyna, Ekaterina Gromova, Natalia Mshenskaya, Anastasiia Ryabkova, Nikolay Ilin, Vladimir Vodeneev, Evgeny Mareev, Colin Price

**Affiliations:** 1 Department of Biophysics, N.I. Lobachevsky State University of Nizhny Novgorod, 603950 Nizhny Novgorod, Russia; n.catherine@inbox.ru (E.S.); kater333@inbox.ru (E.G.); nastay2903@bk.ru (A.R.); v.vodeneev@mail.ru (V.V.); 2 Earth’s Electromagnetic Environment Laboratory, Institute of Applied Physics of Russian Academy of Sciences, 603600 Nizhny Novgorod, Russia; jsin@inbox.ru (Y.S.); tasya.mshanka@yandex.ru (N.M.); ilyin@appl.sci-nnov.ru (N.I.); evgeny.mareev@gmail.com (E.M.); cprice@flash.tau.ac.il (C.P.); 3 Department of Biochemistry and Biotechnology, N.I. Lobachevsky State University of Nizhny Novgorod, 603950 Nizhny Novgorod, Russia; 4 Department of Geophysics, Porter School of the Environment and Earth Sciences, Tel Aviv University, Tel Aviv-Yafo 6997801, Israel

**Keywords:** extremely low frequency magnetic field, Schumann resonance frequencies, photosynthetic light reactions, non-photochemical quenching, quantum yield of photosystem II, wheat, pea

## Abstract

Photosynthesis is an important target of action of numerous environmental factors; in particular, stressors can strongly affect photosynthetic light reactions. Considering relations of photosynthetic light reactions to electron and proton transport, it can be supposed that extremely low frequency magnetic field (ELFMF) may influence these reactions; however, this problem has been weakly investigated. In this paper, we experimentally tested a hypothesis about the potential influence of ELFMF of 18 µT intensity with Schumann resonance frequencies (7.8, 14.3, and 20.8 Hz) on photosynthetic light reactions in wheat and pea seedlings. It was shown that ELFMF decreased non-photochemical quenching in wheat and weakly influenced quantum yield of photosystem II at short-term treatment; in contrast, the changes in potential and effective quantum yields of photosystem II were observed mainly under chronic action of ELFMF. It is interesting that both short-term and chronic treatment decreased the time periods for 50% activation of quantum yield and non-photochemical quenching under illumination. Influence of ELFMF on pea was not observed at both short-term and chronic treatment. Thus, we showed that ELFMF with Schumann resonance frequencies could influence photosynthetic light processes; however, this effect depends on plant species (wheat or pea) and type of treatment (short-term or chronic).

## 1. Introduction

Photosynthesis, participating in solar energy consumption and its transformation into energy of chemical compounds, is a key process in plant life; however, it is also an important target of action of environmental factors and, thereby, can participate in the development of stress changes in plants. There are numerous investigations, which show that photosynthetic processes can be affected by action of environmental factors on plant organism (light with excess intensity [1,2,3], low or high temperatures [4,5,6], drought [7,8,9], salinity [10,11,12], etc.) or by propagation of long-distance stress signals through plant body (e.g., electrical signals [13,14,15]). These photosynthetic changes have intricate character including photosynthetic damage and decrease of photosynthetic intensity (e.g., decrease of CO_2_ assimilation and quantum yield of photosystem II (Φ_PSII_)) [3,9,12] and induction of numerous adaptive responses, which participate in photosynthetic protection and can also modify photosynthetic activity (e.g., increase of non-photochemical quenching (NPQ) [12,16,17], activation of cyclic electron flow [8,17], changes in localization of ferredoxin-NADP-reductase [18,19], etc.).

Photosynthetic light reactions are strongly related to transport of electrons through chloroplast’s electron transport chain (ETC) [16,17] and ion fluxes through thylakoid membranes [20,21]; considering that both processes are charge transfer, they can probably be influenced by magnetic fields (MFs). MFs are important physical factors of environment; they include stationary geomagnetic fields, stationary artificial magnetic fields, and non-stationary MFs. In particular, extremely low frequency magnetic fields (ELFMFs) are widely produced as a result of direct human activity (mainly, MFs with industrial frequencies, equaling to 50 and 60 Hz) and as result of natural environmental events, mainly magnetospheric substorms and lightning [22]. From the beginning of its development, the Earth’s biosphere has been influenced by various electromagnetic fields, among which a special place is occupied by fields at frequencies of Schumann resonances formed by the Earth–ionosphere resonator [22]. The Schumann resonance frequencies equal 7.8, 14.3, 20.8, 27.3, and 33.8 Hz [23,24].

It is important that ELFMFs can strongly influence physiological processes in living organisms (e.g., see works [22,25,26]). There are studies [27,28,29,30] which show the influence of these MFs on growth, seed germination, ions transport, tolerance to environmental stressors, and other processes in plants; however, changes in these processes can be controversial. In particular, numerous investigations (see reviews [27,28,29,30]) focus on the influence of ELFMFs on plant growth processes because positive effects can be important for plant agricultural cultivation, in contrast, negative effects should be considered in plant protection. It is shown that ELFMFs [27,28,29,30] mostly stimulate plant growth and production of biomass; however, their suppression is also observed. Size and (or) direction of the effect is dependent on plant species (e.g., 60 Hz frequency MF strongly stimulates growth of radish, weakly influences the growth in barley and does not influence this process in mustard [31]), duration of long-term ELFMF treatment (e.g., 50 Hz frequency MF stimulates wheat growth at 17–24-h treatment and suppresses it at 2-day treatment [32]), duration of short-term exposition and magnitude of the magnetic fields (e.g., 50 Hz frequency MF induces different growth stimulation at 5, 10, and 15 min of exposition [33]), etc. It is interesting that growth changes can be also observed under low frequencies and magnitudes of MFs; e.g., sinusoidal ELFMF with about 16 Hz frequency and 20 µT magnitude increases root dry weights in wheat and does not influence this parameter in sunflower seedlings [34]. Considering the influence of ELFMFs on processes of plant growth and production of biomass, it can be expected that these magnetic fields influences photosynthetic processes.

There are a number of works (see reviews [27,28,29,30]), which show the influence of ELFMFs on photosynthesis and related processes. In particular, treatment by ELFMF can change the content of chlorophylls and carotenoids [35,36,37,38], stimulate gene expression of ribulose 1,5-bisphosphate carboxylase/oxygenase small subunit [36], influence photosynthetic CO_2_ assimilation and transpiration [36,38,39]. However, there are only few studies, which are devoted to investigation of the influence of ELFMFs on plant seedlings [36,39]; other works are devoted to the influence of these magnetic fields on seeds [35,37,38]. Results of these works are rather contradictory: Yano et al. [39] show that chronic action of ELFMFs can decrease photosynthetic CO_2_ assimilation while the short-term action of these magnetic fields (2 h) does not influence the assimilation; in contrast, Alemán et al. [36] show that short-term action of ELFMFs (3 min) both suppresses and stimulates net photosynthesis (the type of effect is dependent on the stage of development). There are many potential reasons of these differences, including the study of different plants (radish [39] and *Coffea arabica* [36]) and use of very different magnetic field strengths (50 µT [35] and 2 mT [36]). It should be also noted that both papers [36,39] are devoted to the investigation of only MFs with 50 Hz industrial frequency and do not analyze photosynthetic light reactions.

Thus, the problem of the influence of ELFMFs on photosynthetic processes remains poorly investigated; moreover, investigations of the influence of MFs with Schumann resonance frequencies (7.8, 14.3, 20.8, 27.3, and 33.8 Hz [23,24]) on photosynthetic processes are absent. However, ELFMFs with Schumann resonance frequencies are ubiquitously generated as a result of global lightning activity [22]; amplitude of these natural environmental frequencies can be dependent on global climate changes (i.e., it can be indirectly related to human’s activity). Considering these points, problem of the influence of ELFMFs with Schumann resonance frequencies on photosynthetic processes in plants can be important for plant biology. The present work is devoted to the analysis of the influence of ELFMFs with Schumann resonance frequencies (the first, second, and third harmonics that equal 7.83, 14.3, and 20.8 Hz) on photosynthetic light reactions in seedling of wheat and pea.

## 2. Materials and Methods

### 2.1. Materials

Wheat (*Triticum aestivum* L., cultivar “Zlata”) and pea (*Pisum sativum* L., cultivar “Albumen”) seedlings were used in experiments with treatment by magnetic fields. Seeds were soaked for 3 days before planting. Plants were cultivated (up to 9–13-days age) in vegetation room in pots with soil at 24 °C and 16/8 h (light/dark) photoperiod; luminescent lamps FSL YZ18RR (Foshan Electrical And Lighting Co., Ltd., Foshan, China) were used for illumination.

### 2.2. Short-Term and Chronic Treatments by Magnetic Fields with Schumann Resonance Frequencies

**Figure 1:**
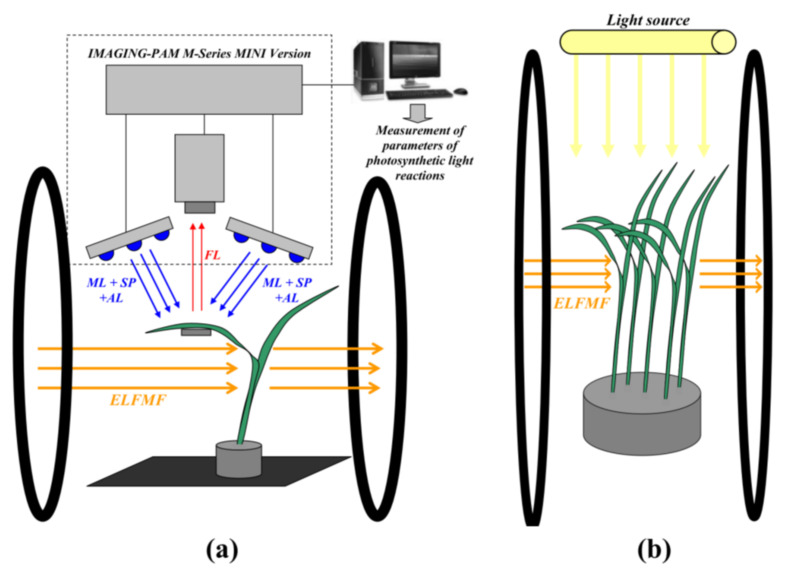
(**a**) Schema of plant localization in experiments with simultaneous action of artificial extremely low frequency magnetic field (ELFMF) and measurements of parameters of photosynthetic light reactions using IMAGING-PAM M-Series MINI Version. ML measuring light, SP saturation pulse, and AL actinic light. Blue light (450 nm) was used for ML, SP, and AL. FL is chlorophyll fluorescence. (**b**) Schema of localization of plants in experiments with chronic action of ELFMF. Luminescent lamps FSL YZ18RR were used as a light source for growth. Wheat plants were used as examples in both figures. Localization of plants in control experiments was identical for both variants; however, they were not treated by ELFMF. The direction of ELFMF was perpendicular to the direction of the geomagnetic field (about 50 µT).

Two systems for plant treatment by artificial ELFMFs were manufactured: the first system was used for photosynthetic measurements in leaves of plants under simultaneous treatment by MFs (volume of homogenous magnetic field was about 20 × 20 × 20 cm^3^) (Figure 1a); the second system was used for plant cultivation under chronic action of ELFMFs (volume of homogenous magnetic field was about 30 × 30 × 30 cm^3^) (Figure 1b). Both systems were based on Helmholtz coils (100 loops for first system and 130 loops for second system for each coil with corrected input impedance of 50 ohm) with different radii (0.3 m for the first system and 0.5 m for the second system). Positions of Helmholtz coils supported the direction of ELFMFs, which was perpendicular to the direction of a geomagnetic field (about 50 µT). RIGOL DG1032 Waveform Generator (RIGOL Technology Co., Ltd., Suzhou, China) was used for the generation of sinusoidal electrical signals with frequencies equaling 7.8, 14.3, and 20.8 Hz (for experiments with short-term treatment) or 14.3 Hz (for experiments with chronic treatment). Magnitude of ELFMFs was 18 µT in all variants of the experiments.

Short-term treatment of wheat seedlings by ELFMFs (in 9–13 days after pea planting and in 9–11 days after wheat planting) was initiated after fixation of plants in the first system; treatment was continued during the measurement of parameters of photosynthetic light reactions (30 min) (Figure 1a). In the control variant, seedlings were fixed in the system and their photosynthetic parameters were measured; however, the ELFMFs did not act on plants.

Both systems were used for chronic treatment of seedlings by the investigated ELFMF: the plants were cultivated in the second system with MF treatment (Figure 1b) from initiation of soaking of seeds to plant planting (3 days) and from plant planting to transfer of seedlings into the first system for photosynthetic measurements (on the 9th day of cultivation) (Figure 1a). The time taken for plant translocation from the second system to the first one was less than 30 s. Parameters of ELFMF in the first system were identical to the ones in the second system in this experimental variant (chronic treatment by MF). Photosynthetic parameters were measured by the action of this field. In control, seedlings were cultivated and measured under similar conditions (analogical system for plant growth was used); however, ELFMF treatment was absent.

### 2.3. Measurements of Parameters of Photosynthetic Light Reactions

All measurements of photosynthetic parameters (excluding control variants) were performed simultaneously with the plant treatment by magnetic fields; duration of the photosynthetic measurements was about 30 min. Only one photosynthetic measurement was performed for each seedling. In experiments with chronic action of ELFMF, seedlings were measured on the 9th day after plant planting; 9–11 days age wheat and 9–13 days age pea seedlings were used for photosynthetic measurements in experiments with short-term action of ELFMFs.

**Figure 2:**
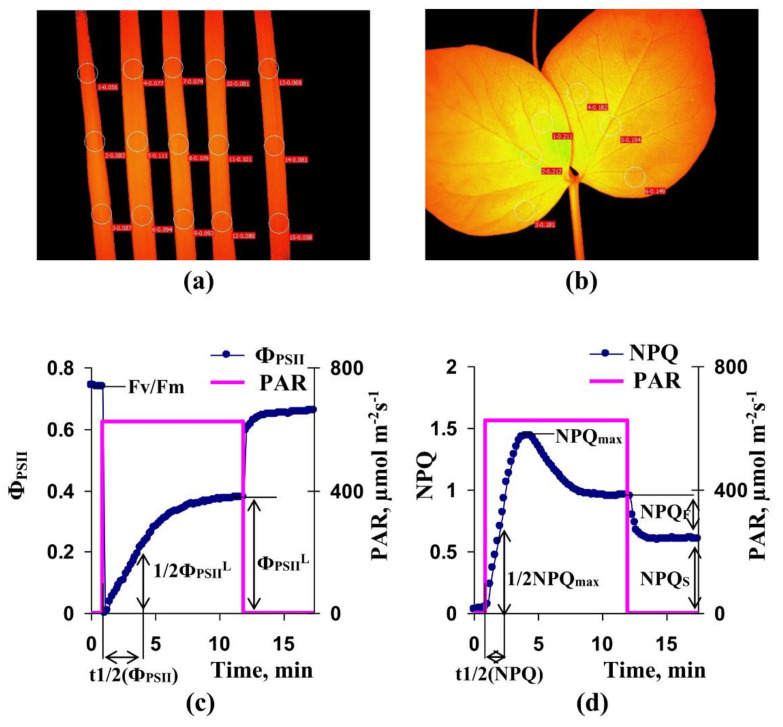
(**a**) Localizations of investigated areas (ROIs) in wheat leaves at PAM-imaging. Photosynthetic parameters were averaged for each wheat leaf (3 ROIs were averaged). (**b**) Localizations of investigated areas (ROIs) in pea leaves at PAM-imaging. Photosynthetic parameters were averaged for pea leaf (6 ROIs were averaged). (**c**) Record of changes in quantum yield of photosystem II (Φ_PSII_) under action of actinic light (its intensity is marked as PAR). Fv/Fm is the potential quantum yield of photosystem II, Φ_PSII_^L^ is the effective quantum yield of photosystem II after 10 min of illumination by actinic light, and t_1/2_(Φ_PSII_) is the time taken for 50% increase of Φ_PSII_ under illumination. Wheat leaf is used for this record. (**d**) Record of changes in non-photochemical quenching (NPQ) under action of actinic light. NPQ_F_ is fast-relaxing component of NPQ after 10 min of illumination, NPQ_S_ is slow-relaxing component of NPQ after this illumination, NPQ_max_ is maximal value of NPQ, and t_1/2_(NPQ) is time taken for 50% increase of NPQ under illumination. Wheat leaf is used for this record.

A system of pulse-amplitude-modulation (PAM) fluorescence imaging (IMAGING-PAM M-Series MINI Version, Heinz Walz GmbH, Effeltrich, Germany) was used for measurements of parameters of photosynthetic light reactions in the second mature leaves of wheat and pea seedlings (Figure 1a). The pulses of measuring light (ML) with low average intensity (less than 1 µmol m^−2^s^−1^), saturation pulses (SP) with 800 ms duration and 6000 µmol m^−2^s^−1^ intensity, and actinic light (AL) with 625 µmol m^−2^s^−1^ intensity were used for analysis. Only, blue light (450 nm) was used for the photosynthetic investigations. Five wheat leaves were simultaneously investigated in each experiment; photosynthetic parameters were calculated as averaging of ones in three areas in each leaf (Figure 2a). Single pea leaf was investigated in each experiment; photosynthetic parameters were calculated as averaging of ones in six areas in the leaf (Figure 2b). Standard round areas (which were formed by software of IMAGING-PAM) were used; their localizations were approximately similar for all investigated leaves in the control and ELFMF-treated plants. The procedure minimized errors, which could be related by different shapes and areas of the investigates leaves.

Seedlings were adapted to dark conditions for 15 min after fixation in the measuring system. After that, ML was turned on and SPs were periodically generated every 10 s. The first SP was used for estimation of initial and maximum rates of photosystem II fluorescence (*F*_0_ and *F_m_*, respectively); following SPs were used for estimation of the current rates of fluorescence (*F*) and maximum fluorescence rates under light conditions (*F_m_*′). AL was turned on for 80 s after the first SP; duration of illumination by actinic light was about 10 min. Periodical SPs were generated for 5 min after termination of illuminations. Parameters of photosystem II, including Fv/Fm, the potential quantum yield of photosystem II, Φ_PSII_, the effective quantum yield of photosystem II, and NPQ, the non-photochemical quenching of chlorophyll fluorescence, were calculated on the basis of *F*_0_, *F_m_*, *F*, and *F_m_*′ in accordance to standard equations [16,40,41].

For further analysis, we used Fv/Fm, Φ_PSII_^L^ (effective quantum yield of photosystem II after 10 min of illumination by actinic light), and t_1/2_(Φ_PSII_) (time taken for 50% increase of Φ_PSII_ under illumination); estimation of these parameters are shown in Figure 2c. In addition, we used NPQ_F_ (fast-relaxing component of NPQ after 10 min of illumination), NPQ_S_ (slow-relaxing component of NPQ after this illumination), NPQ_max_ (maximal value of NPQ during illumination), and t_1/2_(NPQ) (time taken for 50% increase of NPQ under illumination); estimation of these parameters were shown in Figure 2d.

### 2.4. Statistics

We used 30 wheat seedlings and 9 pea seedlings for each variant of the experiment (control, 7.8, 14.3, and 20.8 Hz) with short-term treatments by magnetic fields. 30 Wheat seedlings and 6 pea seedlings were exposed to chronic treatment by ELFMF (14.3 Hz); same quantities of plants were used in the control. Mean values, standard errors, and Pearson’s correlation coefficients were presented. The Student’s t-test was used for estimation of significance of differences between control plants and plants treated by ELFMFs.

## 3. Results

### 3.1. Investigation of the Influence of Short-Term Treatment by Magnetic Fields with Schumann Resonance Frequencies on Parameters of Photosynthetic Light Reactions

Investigation of the influence of short-term treatment by magnetic fields with Schumann resonance frequencies (the first, second, and third harmonics) on parameters of photosynthetic light reactions in wheat and pea seedlings was the first task of our work.

**Figure 3:**
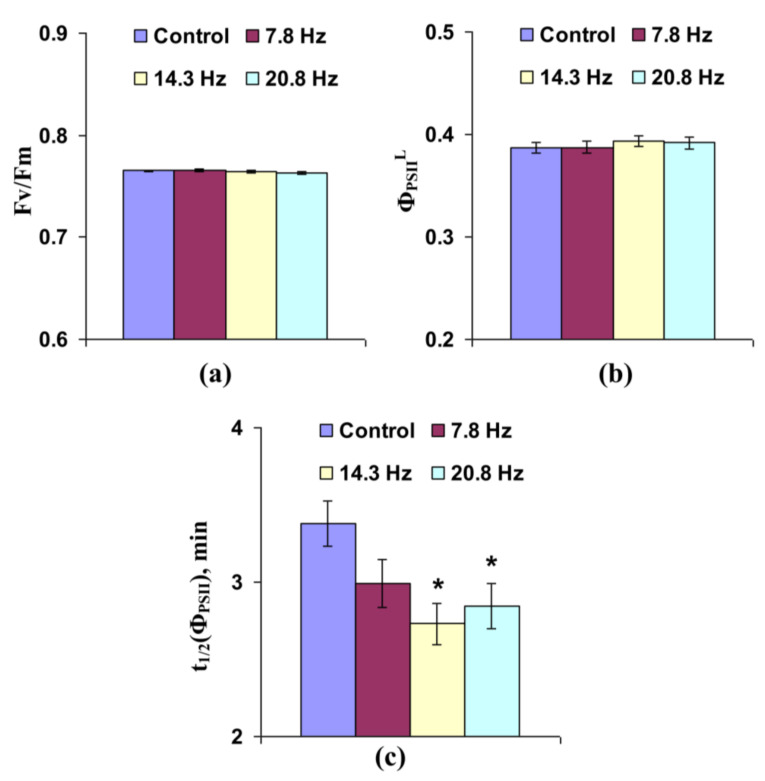
Influence of short-term treatment by artificial extremely low frequency magnetic field on potential quantum yield of photosystem II (Fv/Fm) (**a**), effective quantum yield of photosystem II under illumination (Φ_PSII_^L^) (**b**), and time taken for 50% increase of Φ_PSII_ under illumination (t_1/2_(Φ_PSII_)) (**c**) in wheat seedlings (*n* = 30). Action of the artificial magnetic field was immediately initiated before dark adaptation; total duration of its action was 30 min. Photosynthetic parameters were measured by the action of this field. Magnitude of the magnetic fields was 18 µT; frequencies were 7.8, 14.3, and 20.8 Hz. Control plants were not treated by this artificial magnetic field. *, difference between the experiment and control plants was significant (*p* < 0.05).

Figure S1 shows light-induced changes in Φ_PSII_ and NPQ, which were different in the control plants and those treated by ELFMF. Figure 3a shows that short-term treatment by ELFMFs (with frequencies equaling to 7.8, 14.3, and 20.8 Hz) did not influence the potential quantum yield of photosystem II, which was related to the maximal photochemical efficiency of photosystem II [16,40,41], in leaves of wheat seedlings. Effective quantum yield under illumination by AL, which was related to the photochemical efficiency of photosystem II under used light conditions [16,40,41], was also not affected by ELFMFs (Figure 3b). In contrast, significant decrease in time taken for 50% increase of Φ_PSII_ under illumination, which can likely be related to light-induced activation of ETC [42,43], was observed under action of MFs with 14.3 and 20.8 Hz frequencies (Figure 3c). This decrease was not significant under the action of ELFMF with 7.8 Hz frequency; however, the tendency for this decrease was also observed at this variant of MF treatment.

**Figure 4:**
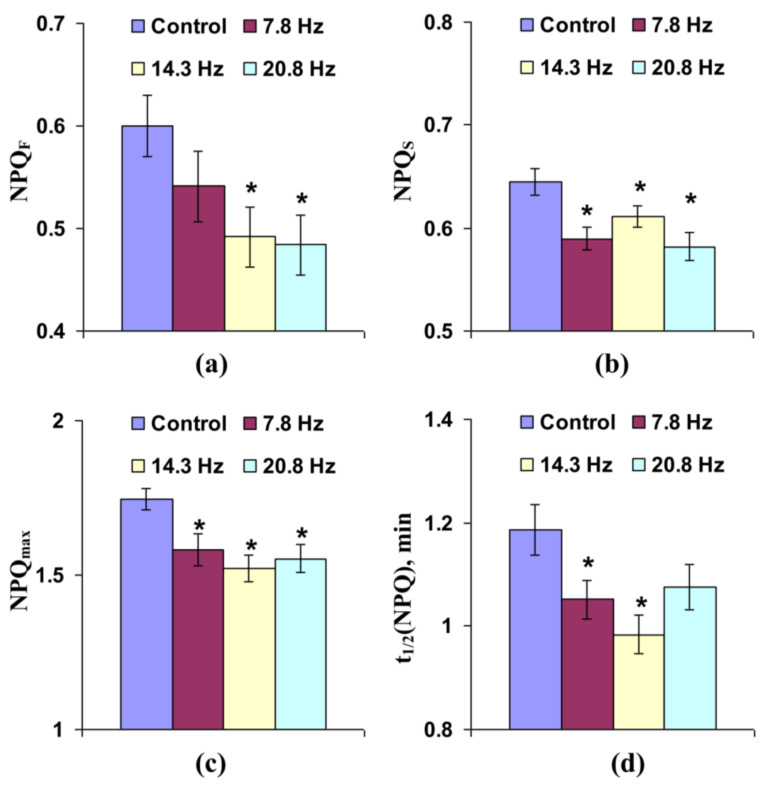
Influence of short-term treatment by artificial extremely low frequency magnetic field on the fast-relaxing component of non-photochemical quenching under illumination (NPQ_F_) (**a**), slow-relaxing component of non-photochemical quenching after this illumination (NPQ_S_) (**b**), maximal value of non-photochemical quenching (NPQ_max_) (**c**), and time taken for 50% increase of NPQ under illumination (t_1/2_(NPQ)) (**d**) in wheat seedlings (*n* = 30). Action of the artificial magnetic field was immediately initiated before dark adaptation; total duration of its action was 30 min. Photosynthetic parameters were measured by the action of this field. Magnitude of the magnetic field was 18 µT; frequencies were 7.8, 14.3 and 20.8 Hz. Control plants were not treated by this artificial magnetic field. *, difference between the experiment and control plants was significant (*p* < 0.05).

Influence of short-term treatment by the investigated ELFMFs on non-photochemical quenching of chlorophyll fluorescence was more expressive. It was shown (Figure 4a) that the treatment of wheat seedlings by MFs with 14.3 and 20.8 Hz frequency significantly decreased fast-relaxing component of NPQ, which can be considered as an energy-dependent component of the non-photochemical quenching [40,43,44]. Figure 4b shows that treatment by ELFMFs with frequencies equaling 7.8, 14.3, and 20.8 Hz could also decrease the slow-relaxing component of non-photochemical quenching, which was probable to be related to components of NPQ, which were caused by state transition (migration of the light harvesting complex II from photosystem II to photosysthem I) and photodamage of photosystem II [3,40,43,45,46]. Figure 4c shows that all used frequencies of ELFMFs, which were used for short-term treatment, decreased the maximal value of NPQ. Figure 4d shows that ELFMFs with 7.8 and 14.3 Hz frequencies decreased time taken for 50% increase of NPQ under illumination. The 20.8 Hz magnetic field also decreased this NPQ activation time; however, the effect was not significant. Considering the strong relation between light-induced photosynthetic activation and transient increase of NPQ (e.g., see our theoretical work [43]), decreases of both NPQ_max_ and t_1/2_(NPQ) could reflect stimulation of electron transport through ETC and acceleration of H^+^ transport through thylakoid membranes.

Thus, results of analysis of the influence of short-term treatment by ELFMFs with frequencies equaling 7.8, 14.3, and 20.8 Hz on photosynthetic light reactions in leaves of wheat seedlings showed that these MFs could modify photosystem II parameters (especially, NPQ). This effect was most stable under MFs of 14.3 Hz frequency; as a result, we supposed that ELFMF with this frequency could be used for the analysis of the action of chronic treatment of wheat on photosynthetic light reactions.

**Figure 5:**
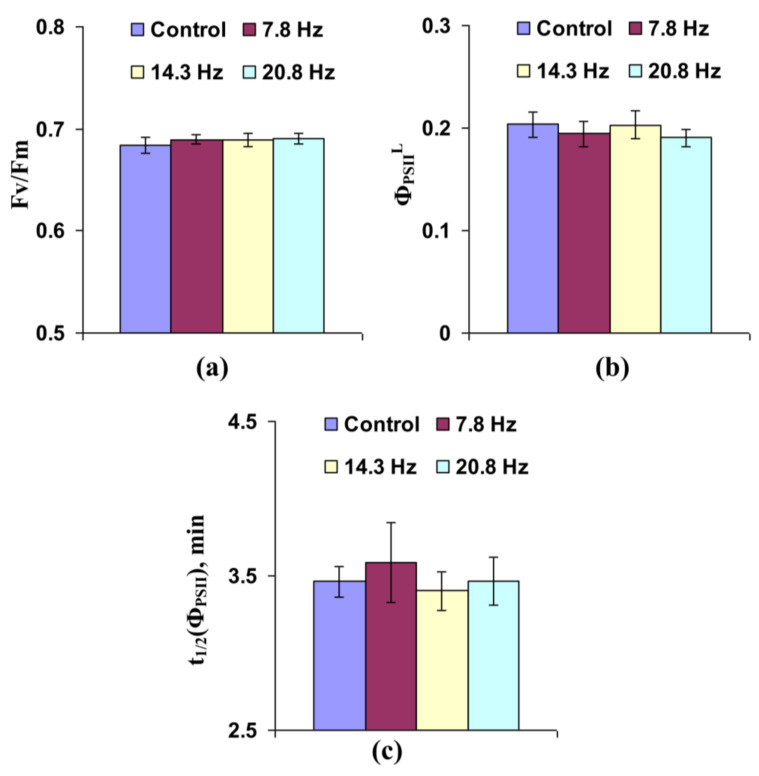
Influence of short-term treatment by artificial extremely low frequency magnetic field on potential quantum yield of photosystem II (Fv/Fm) (**a**), effective quantum yield of photosystem II under illumination (Φ_PSII_^L^) (**b**), and time taken for 50% increase of Φ_PSII_ under illumination (t_1/2_(Φ_PSII_)) (**c**) in pea seedlings (*n* = 9). Action of the artificial magnetic field was immediately initiated before dark adaptation; total duration of its action was 30 min. Photosynthetic parameters were measured by the action of this field. Magnitude of the magnetic fields was 18 µT; frequencies were 7.8, 14.3 and 20.8 Hz. Control plants were not treated by this artificial magnetic field. Significant differences between the experiment and control plants were absent.

**Figure 6:**
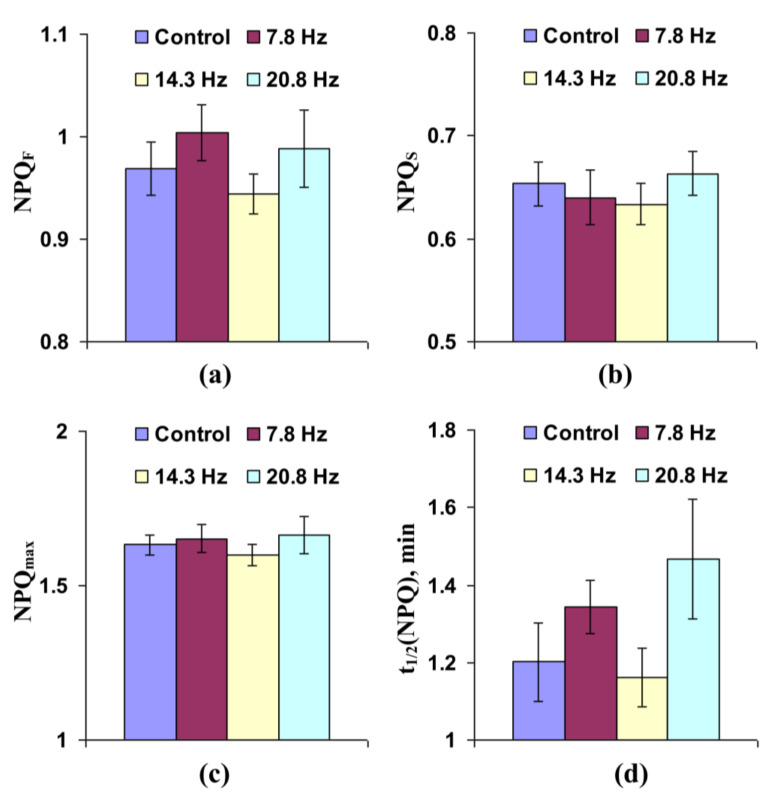
Influence of short-term treatment by artificial extremely low frequency magnetic field on fast-relaxing component of non-photochemical quenching under illumination (NPQ_F_) (**a**), slow-relaxing component of non-photochemical quenching after this illumination (NPQ_S_) (**b**), maximal value of non-photochemical quenching (NPQ_max_) (**c**), and time taken for 50% increase of NPQ under illumination (t_1/2_(NPQ)) (**d**) in pea seedlings (*n* = 9). Action of the artificial magnetic field was immediately initiated before dark adaptation; total duration of its action was 30 min. Photosynthetic parameters were measured by the action of this field. Magnitude of the magnetic fields was 18 µT; frequencies were 7.8, 14.3, and 20.8 Hz. Control plants were not treated by this artificial magnetic field. Significant differences between the experiment and control plants were absent.

Analysis of the influence of short-term treatment by ELFMFs with 7.8, 14.3, and 20.8 Hz frequencies on photosynthetic light reactions in leaves of pea seedlings showed other results. Figure S2 shows light-induced changes in Φ_PSII_ and NPQ, which seemed to be similar in the control plants and those treated by ELFMF. Figure 5 and Figure 6 show that this treatment did not influence all photosynthetic parameters, which were investigated in our work (Fv/Fm, Φ_PSII_^L^, t_1/2_(Φ_PSII_), NPQ_F_, NPQ_S_, NPQ_max_, and t_1/2_(NPQ)); even tendencies to changes were absent. As a result, we could not select an optimal frequency for analysis of the chronic action of ELFMFs on pea seedlings on the basis of these results. Considering the high efficiency of short-term treatment of wheat seedlings by 14.3 Hz MFs, we also used the second harmonic in Schumann resonance frequencies for investigation of the influence of chronic ELFMF on pea seedlings in a further investigation.

### 3.2. Investigation of the Influence of Chronic Treatment by Magnetic Fields with the Second Harmonic in Schumann Resonance Frequencies on Parameters of Photosynthetic Light Reactions

**Figure 7:**
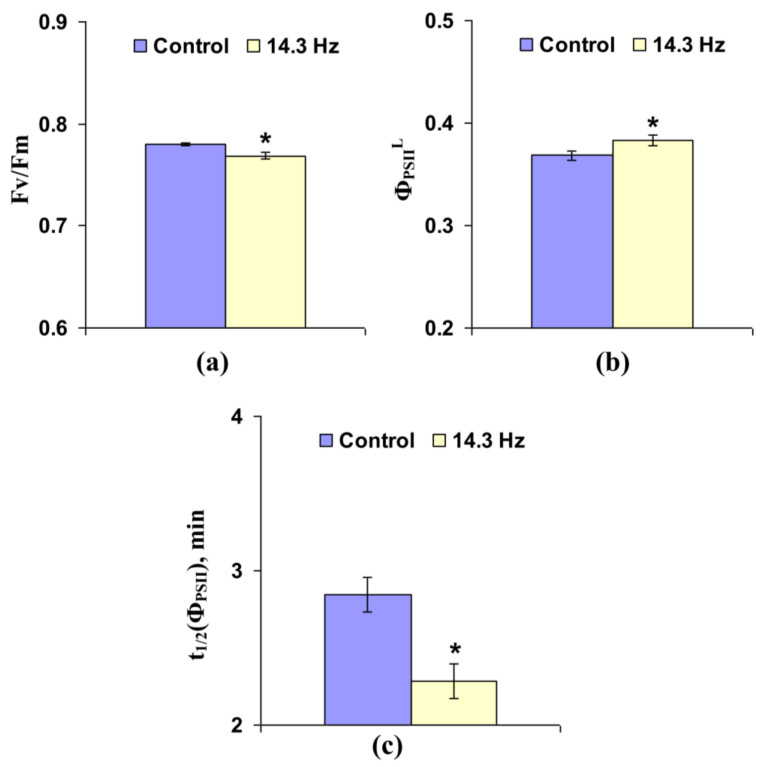
Influence of chronic treatment by artificial extremely low frequency magnetic field on potential quantum yield of photosystem II (Fv/Fm) (**a**), effective quantum yield of photosystem II under illumination (Φ_PSII_^L^) (**b**), and time taken for 50% increase of Φ_PSII_ under illumination (t_1/2_(Φ_PSII_)) (**c**) in wheat seedlings (*n* = 30). Chronic action of the artificial magnetic field was initiated from soaking of seeds. Magnitude of the magnetic field was 18 µT; frequency was 14.3 Hz. Photosynthetic parameters were measured by the action of this field. Control plants were not treated by this artificial magnetic field. *, difference between the experiment and control plants was significant (*p* < 0.05).

Analysis of the influence of chronic treatment by ELFMF with 14.3 Hz frequency on photosynthetic light reactions in leaves of wheat seedlings showed that the effect of this treatment was different from the effect of short-term treatments by the investigated ELFMFs (Figure 7 and Figure S3). The potential quantum yield of photosystem II decreased under chronic treatment by 14.3 Hz frequency MF (Figure 7a); in contrast, the effective quantum yield of photosystem II under light conditions was increased under this treatment (Figure 7b). It is important that magnitudes of both changes were small (about 1.5% for Fv/Fm and 4% for Φ_PSII_^L^); these magnitudes were lower than photosynthetic changes, which were induced by short-term ELFMFs treatment (mainly, 10–20%). Figure 7c shows that time taken for 50% increase of Φ_PSII_ under illumination decreased under chronic action of 14.3 Hz frequency MF; magnitude of this effect was about 20% and was similar to the magnitudes of photosynthetic changes under short-term action of ELFMFs.

**Figure 8:**
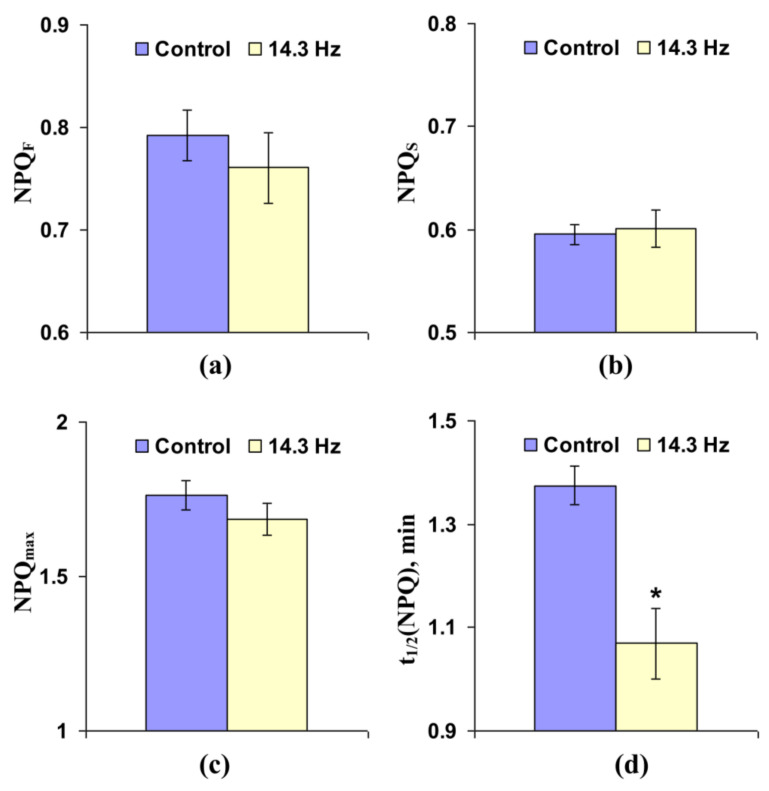
Influence of chronic treatment by artificial extremely low frequency magnetic field on fast-relaxing component of non-photochemical quenching under illumination (NPQ_F_) (**a**), slow-relaxing component of non-photochemical quenching after this illumination (NPQ_S_) (**b**), maximal value of non-photochemical quenching (NPQ_max_) (**c**), and time taken for 50% increase of NPQ under illumination (t_1/2_(NPQ)) (**d**) in wheat seedlings (*n* = 30). Chronic action of the artificial magnetic field was initiated from soaking of seeds. Magnitude of the magnetic field was 18 µT; frequency was 14.3 Hz. Photosynthetic parameters were measured by the action of this field. Control plants were not treated by this artificial magnetic field. *, difference between the experiment and control plants was significant (*p* < 0.05).

Figure 8 shows that chronic treatment by 14.3 Hz frequency ELFMF did not change the fast and slow relaxing components of NPQ and maximal value of non-photochemical quenching in leaves of wheat seedlings; in contrast, time taken for 50% increase of NPQ under illumination was significantly decreased by this chronic MF treatment (more than 20%).

**Figure 9:**
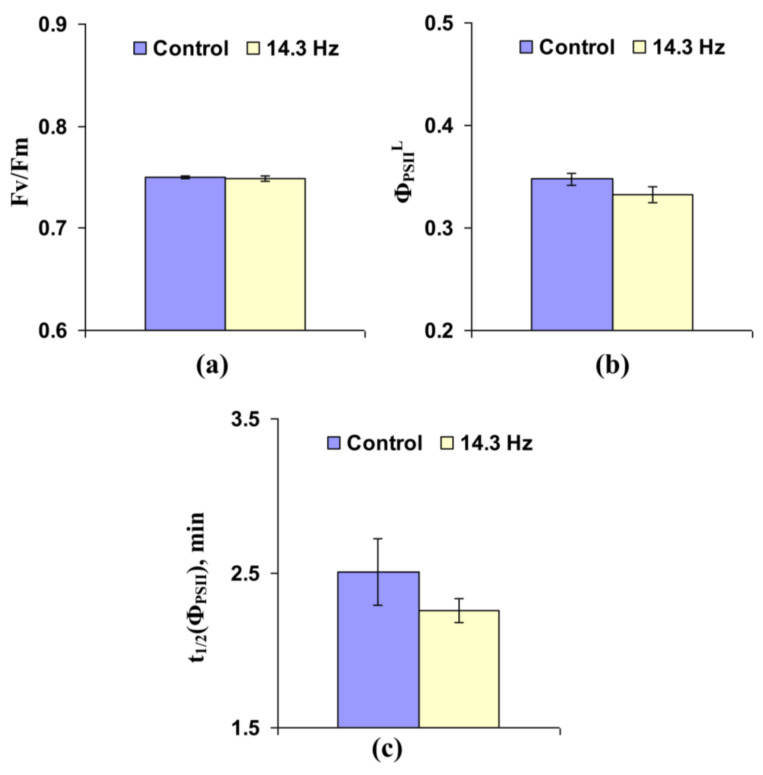
Influence of chronic treatment by the artificial extremely low frequency magnetic field on the potential quantum yield of photosystem II (Fv/Fm) (**a**), effective quantum yield of photosystem II under illumination (Φ_PSII_^L^) (**b**), and time taken for 50% increase of Φ_PSII_ under illumination (t_1/2_(Φ_PSII_)) (**c**) in pea seedlings (*n* = 6). Chronic action of the artificial magnetic field was initiated from soaking of seeds. Magnitude of the magnetic field was 18 µT; frequency was 14.3 Hz. Photosynthetic parameters were measured by the action of this field. Control plants were not treated by this artificial magnetic field. Significant differences between the experiment and control plants were absent.

**Figure 10:**
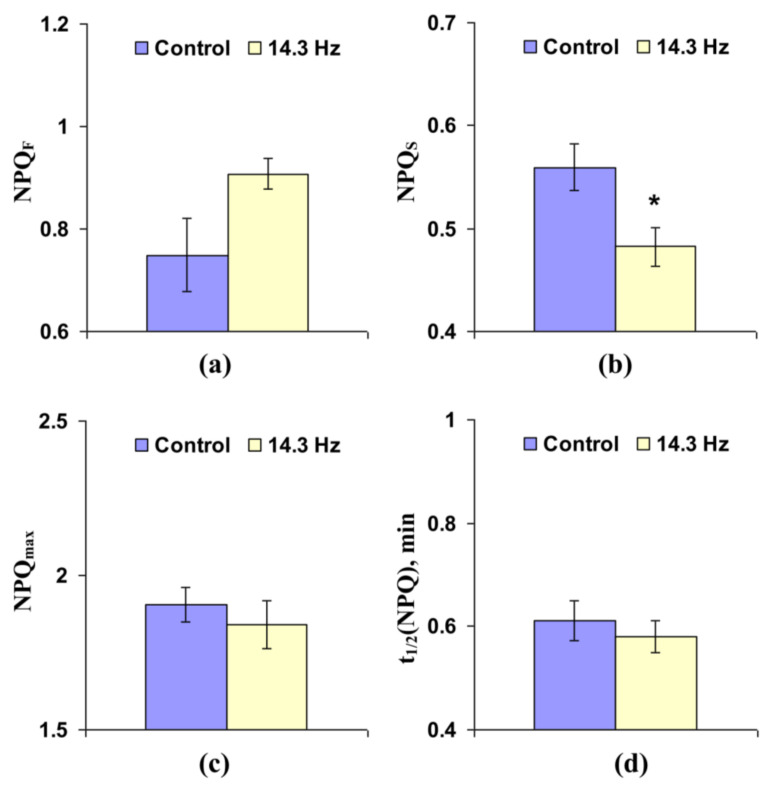
Influence of chronic treatment by the artificial extremely low frequency magnetic field on fast-relaxing component of non-photochemical quenching under illumination (NPQ_F_) (**a**), slow-relaxing component of non-photochemical quenching after this illumination (NPQ_S_) (**b**), maximal value of non-photochemical quenching (NPQ_max_) (**c**), and time taken for 50% increase of NPQ under illumination (t_1/2_(NPQ)) (**d**) in pea seedlings (*n* = 6). Chronic action of the artificial magnetic field was initiated from soaking of seeds. Magnitude of the magnetic field was 18 µT; frequency was 14.3 Hz. Photosynthetic parameters were measured by the action of this field. Control plants were not treated by this artificial magnetic field. *, difference between the experiment and control plants was significant (*p* < 0.05).

Figure 9, Figure 10, and Figure S4 show that chronic action of 14.3 Hz frequency ELFMF did not influence most of the investigated parameters of photosynthetic light reactions in leaves of peas seedlings, excluding the slow-relaxing component of NPQ. This parameter was significantly decreased under chronic action of ELFMF with frequency equaling 14.3 Hz (Figure 10b); the magnitude of this effect was about 14%. It is also interesting that a weak tendency to decrease under chronic treatment by 14.3 Hz frequency ELFMF was observed for t_1/2_(Φ_PSII_) and t_1/2_(NPQ). This effect was similar to changes in these parameters in wheat seedlings under short-term and chronic treatment by MFs.

## 4. Discussion

It is known that photosynthesis can be strongly affected by numerous environmental stressors, including light with excess intensity [1,2,3], low or high temperatures [4,5,6], drought [7,8,9], salinity [10,11,12] or long-distance signals, relating to the action of stressors (e.g., electrical signals [13,14,15]). However, there are environmental factors with weakly-investigated influence on photosynthetic processes; in particular, ELFMFs that can be produced by direct human activity (mainly, 50 and 60 Hz [22]) and by natural environmental events (mainly, thunderstorm-caused Schumann resonance frequencies [23,24,25]). Considering the strong relation of photosynthetic activity to electron transport through chloroplast’s ETC [16,17] and ion fluxes through thylakoid membranes [20,21], it can be expected that these processes should be affected by EFLMFs, which can likely influence charge transfer in different ways [27].

There is a small amount of work devoted to the investigation of ELFMFs on photosynthesis and relating processes (see reviews [27,28,29,30]). Moreover, only few works show the influence of these MFs on photosynthesis in plant seedlings [36,39]; other works investigate the influence on photosynthetic parameters in plants after seed treatments by ELFMFs [35,37,38]. Our current work, which is devoted to the analysis of the influence of ELFMFs with 7.8, 14.3, and 20.8 Hz frequencies (the first, second and third Schumann resonance frequencies) on parameters of photosynthetic light reactions, shows the following points:

1. ELFMFs with Schumann resonance frequencies can significantly influence photosynthetic parameters in plants; however, this effect is dependent on plant species: changes are observed in wheat seedlings (Figure 3, Figure 4, Figure 7 and Figure 8) and mainly absent in pea seedlings (Figure 5, Figure 6, Figure 9 and Figure 10), excluding NPQ_S_ decrease under chronic action of MF (Figure 10b).

2. Effects induced by short-term and chronic treatments by ELFMFs are different: short-term action mainly influences NPQ (Figure 4a–c), chronic action mainly modifies quantum yields (Figure 7a,b). However, magnitudes of changes in these yields under chronic action of MFs (1.5–4%) are much lower than magnitudes of changes in NPQ under the short-term action (10–20%).

3. Both short-term and chronic treatments by ELFMFs are likely to decrease the time of light-induced activation of ETC in wheat seedlings, because decreases of t1/2(Φ_PSII_) (Figure 3c and Figure 7c) and t1/2(NPQ) (Figure 4d and Figure 8d) are shown under short-term and chronic treatments. Relative magnitudes of these decreases are about 20%.

The last point shows some potential mechanisms of revealing photosynthetic changes. t1/2(NPQ) for wheat seedling is about 0.9–1.4 min in our experiments (Figure 4d and Figure 8d). These fast changes are related to the induction of the energy-dependent component of NPQ, which is caused by pH decrease in the chloroplast lumen and protonation of PsbS proteins in the light-harvesting complex [46,47,48]; i.e., they are dependent on proton transport through a thylakoid membrane. It should be additionally noted that slower mechanisms of NPQ can also be dependent on proton transport: synthesis of zeaxanthin and anteraxanthin from violaxanthin in the xanthophyll cycle [46,49,50,51] and state transition [52] are activated by decrease of pH in lumen.

It is probable that light-induced increase of Φ_PSII_ (Figure 3c, Figure 7c, and Figures S1 and S3) is also influenced by proton transport through thylakoid membrane and increased pH in the stroma of chloroplasts [53] because stroma alkalization can stimulate enzymes of the Calvin-Benson cycle [54,55] and activate ferredoxin-NADP-reductase through change in its localization [18,19].

If the hypothesis about participation of proton transport in both NPQ and Φ_PSII_ increase under illumination is correct, it should be expected that t_1/2_(NPQ) and t_1/2_(Φ_PSII_) should be related. Correlation analysis shows that Pearson’s correlation coefficients between these values are 0.71 (*p* < 0.05) and 0.68 (*p* < 0.05) for the control wheat seedlings in short-term and chronic experiments, respectively. Moreover, these coefficients are 0.61–0.78 for different experimental variants with treatment by MFs (*p* < 0.05 for all variants, data not shown). The results support the hypothesis about participation of proton transport in the increase of NPQ and Φ_PSII_ under illumination.

There are different mechanisms of proton transport through thylakoid membranes [20,21,56]: proton influx to lumen at ETC activity, proton efflux to stroma through H^+^-ATP-synthase, and proton fluxes through co-transporters of protons and ions (in particular, Ca^2+^/H^+^ antiport in thylakoid membrane [57]). Potentially, increases in rates of both H^+^ influx to lumen and H^+^ efflux to stroma should decrease time of forming of stationary pH; however, increase in H^+^ influx is accompanied by stimulation of lumen acidification under illumination and increase of H^+^ efflux is related to weakening of this acidification. Our experimental results show that NPQ_F_, NPQ_S_, and NPQ_max_ are decreased in wheat seedlings under short-term treatment by ELFMFs, which induces a decrease of t_1/2_(NPQ) and t_1/2_(Φ_PSII_) (Figure 4); moreover, similar insignificant decreases are also observed under chronic action of ELFMFs (Figure 8). Considering a strong relation between NPQ formation and decrease of pH in lumen [46,47,48,49,50,51,52], the results support weakening of lumen acidification under treatment by ELFMFs; i.e., increase of rate of H^+^ efflux is a more probable result of wheat treatment by ELFMFs than increase in H^+^ influx rate.

Probable stimulation of H^+^ efflux by treatment by ELFMFs with Schumann resonance frequencies can also be supported by literature data. (i) Increase in the membrane permeability for ions (including protons) after plant treatment by ELFMFs [58]; the effect is observed at 10 or 100 µT and 50 or 60 Hz. Similar increase in the membrane permeability can contribute to proton leak and stimulate H^+^ efflux to lumen. (ii) ELFMFs can influence Ca^2+^ homeostasis in plants (including increase in free Ca^2+^ concentration) [59,60,61,62,63] that can be related to the direct or indirect effects of cyclotron resonance [27,62]. Considering Ca^2+^/H^+^ antiport in thylakoid membrane [53], increase in Ca^2+^ concentration can stimulate H^+^ efflux. (iii) Increase in Ca^2+^ concentration can also modify the activity of the Calvin-Benson cycle [57,64]; in accordance with our previous work, modification of the photosynthetic dark stage can strongly influence the activity of H^+^-ATP-synthase and luminal and stromal pH [65].

The influence of ELFMFs on photosynthetic light reactions in wheat seedlings and the absence of the influence in pea plants is another important result of our work. It is in good agreement with the results of others (e.g., [31,34]), which show different MF-induced physiological changes in different plant species. Mechanisms of revealed differences in photosynthetic responses on ELFMFs require future investigations; however, some potential ways can be speculated. Our results show that pea seedlings are more sensitive to light intensity than the wheat ones. In particular, pea leaves have low Φ_PSII_ (Figure 5b and Figure 9b) in comparison to wheat leaves (Figure 3b and Figure 7b) under the used intensity of actinic light. The energy-dependent component of NPQ in pea leaves is larger than this parameter in wheat (average NPQ_F_, which is calculated on the basis of all control plants, is 0.881 ± 0.042 in pea and 0.696 ± 0.023 in wheat, *p* < 0.05) and NPQ relaxation under light conditions is slower (Figures S1–S4). Considering the relation between energy-dependent NPQ forming and lumen acidification [46,47,48,49,50,51,52], pea chloroplasts can likely have a larger magnitude of proton gradient across the thylakoid membrane than wheat ones. In this case, the influence of the ELFMF-induced increase of proton leak on pH in lumen should be weak (proton concentration in lumen is high). Additionally, the potential effect of ELFMF-induced stimulation of Ca^2+^/H^+^ antiport, which is localized in the thylakoid membrane [53], can also be weak because high proton gradient should induce strong Ca^2+^ flux into the lumen even without the additional stimulation by ELFMFs (i.e., this additional stimulation of antiporter probably weakly influences Ca^2+^ concentrations in lumen and stroma of chloroplast).

As a whole, we reveal changes in the parameters of photosynthetic light reactions in wheat seedlings under short-term and chronic treatment by ELFMFs with Schumann resonance frequencies. The results show that photosynthetic light reactions can be affected by ELFMFs and that light-induced changes in photosynthetic processes (possibly relating to proton transport through thylakoid membrane) are likely to be more sensitive to the action of these magnetic fields than stationary photosynthetic parameters.

## 5. Conclusions

We investigated the influence of ELFMFs with Schumann resonance frequencies (7.8, 14.3, and 20.8 Hz) on parameters of photosynthetic light reactions in wheat and pea seedlings. These are the following points shown in our current investigation:

ELFMFs with Schumann resonance frequencies can significantly influence photosynthetic parameters in plants; however, this effect is dependent on plant species: changes are observed in wheat seedlings and mainly absent in pea seedlings, excluding NPQ_S_ decrease under chronic action of MF.

Effects induced by short-term and chronic treatments by ELFMFs are different: short-term action mainly influences NPQ, while chronic action mainly modifies quantum yields. However, magnitudes of changes in these yields under chronic action of MFs are much lower than those in NPQ under the short-term action.

Both short-term and chronic treatments by ELFMFs are likely to decrease the time of light-induced photosynthetic activation in wheat seedlings, because decreases in t_1/2_(Φ_PSII_) and t_1/2_(NPQ) are shown under short-term and chronic treatments.

## Data Availability

The data presented in this study are available on request from the corresponding author.

## Supplementary Materials

The following are available online at https://www.mdpi.com/2073-4409/10/1/149/s1, Figure S1: Average dynamics of changes in quantum yield of photosystem II (Φ_PSII_) (a) and non-photochemical quenching (NPQ) (b) under action of actinic light (its intensity is marked as PAR) in wheat seedlings under short-term action of ELFMFs with different frequencies. Figure S2: Average dynamics of changes in quantum yield of photosystem II (Φ_PSII_) (a) and non-photochemical quenching (NPQ) (b) under action of actinic light (its intensity is marked as PAR) in pea seedlings under short-term action of ELFMFs with different frequencies. Figure S3: Average dynamics of changes in quantum yield of photosystem II (Φ_PSII_) (a) and non-photochemical quenching (NPQ) (b) under action of actinic light (its intensity is marked as PAR) in wheat seedlings under chronic action of ELFMF. Figure S4: Average dynamics of changes in quantum yield of photosystem II (Φ_PSII_) (a) and non-photochemical quenching (NPQ) (b) under action of actinic light (its intensity is marked as PAR) in pea seedlings under chronic action of ELFMF.

## References

[1] Quiles M.J., López N.I. (2004). Photoinhibition of photosystems I and II induced by exposure to high light intensity during oat plant growth. Effects on the chloroplast NADH dehydrogenase complex. Plant Sci..

[2] Kalaji H.M., Carpentier R., Allakhverdiev S.I., Bosa K. (2012). Fluorescence parameters as early indicators of light stress in barley. J. Photochem. Photobiol. B.

[3] Murata N., Allakhverdiev S.I., Nishiyama Y. (2012). The mechanism of photoinhibition *in vivo*: Re-evaluation of the roles of catalase, α-tocopherol, non-photochemical quenching, and electron transport. Biochim. Biophys. Acta.

[4] Kanervo E., Tasaka Y., Murata N., Aro E.M. (1997). Membrane lipid unsaturation modulates processing of the photosystem II reaction center protein D1 at low temperatures. Plant Physiol..

[5] Battisti D.S., Naylor R.L. (2009). Historical warnings of future food insecurity with unprecedented seasonal heat. Science.

[6] Allakhverdiev S.I., Kreslavski V.D., Klimov V.V., Los D.A., Carpentier R., Mohanty P. (2008). Heat stress: An overview of molecular responses in photosynthesis. Photosynth. Res..

[7] Lawlor D.W., Tezara W. (2009). Causes of decreased photosynthetic rate and metabolic capacity in water-deficient leaf cells: A critical evaluation of mechanisms and integration of processes. Ann. Bot..

[8] Zivcak M., Brestic M., Balatova Z., Drevenakova P., Olsovska K., Kalaji H.M., Yang X., Allakhverdiev S.I. (2013). Photosynthetic electron transport and specific photoprotective responses in wheat leaves under drought stress. Photosynth. Res..

[9] Urban L., Aarrouf J., Bidel L.P.R. (2017). Assessing the effects of water deficit on photosynthesis using parameters derived from measurements of leaf gas exchange and of chlorophyll a fluorescence. Front. Plant Sci..

[10] Stepien P., Johnson G.N. (2009). Contrasting responses of photosynthesis to salt stress in the glycophyte *Arabidopsis* and the halophyte *Thellungiella*: Role of the plastid terminal oxidase as an alternative electron sink. Plant Physiol..

[11] Mehta P., Kraslavsky V., Bharti S., Allakhverdiev S.I., Jajoo A. (2011). Analysis of salt stress induced changes in Photosystem II heterogeneity by prompt fluorescence and delayed fluorescence in wheat (*Triticum aestivum*) leaves. J. Photochem. Photobiol. B..

[12] Acosta-Motos J.R., Ortuño M.F., Bernal-Vicente A., Diaz-Vivancos P., Sanchez-Blanco M.J., Hernandez J.A. (2017). Plant responses to salt stress: Adaptive mechanisms. Agronomy.

[13] Pavlovič A., Volkov A. (2012). The effect of electrical signals on photosynthesis and respiration. Plant Electrophysiology.

[14] Szechyńska-Hebda M., Lewandowska M., Karpiński S. (2017). Electrical signaling, photosynthesis and systemic acquired acclimation. Front Physiol..

[15] Sukhov V., Sukhova E., Vodeneev V. (2019). Long-distance electrical signals as a link between the local action of stressors and the systemic physiological responses in higher plants. Prog. Biophys. Mol. Biol..

[16] Kalaji H.M., Schansker G., Ladle R.J., Goltsev V., Bosa K., Allakhverdiev S.I., Brestic M., Bussotti F., Calatayud A., Dąbrowski P. (2014). Frequently asked questions about in vivo chlorophyll fluorescence: Practical issues. Photosynth. Res..

[17] Cruz J.A., Avenson T.J., Kanazawa A., Takizawa K., Edwards G.E., Kramer D.M. (2005). Plasticity in light reactions of photosynthesis for energy production and photoprotection. J. Exp. Bot..

[18] Alte F., Stengel A., Benz J.P., Petersen E., Soll J., Groll M., Bölter B. (2010). Ferredoxin: NADPH oxidoreductase is recruited to thylakoids by binding to a polyproline type II helix in a pH-dependent manner. Proc. Natl. Acad. Sci. USA.

[19] Benz J.P., Stengel A., Lintala M., Lee Y.H., Weber A., Philippar K., Gügel I.L., Kaieda S., Ikegami T., Mulo P. (2010). Arabidopsis Tic62 and ferredoxin-NADP(H) oxidoreductase form light-regulated complexes that are integrated into the chloroplast redox poise. Plant Cell..

[20] Pottosin I., Shabala S. (2016). Transport across chloroplast membranes: Optimizing photosynthesis for adverse environmental conditions. Mol. Plant.

[21] Szabò I., Spetea C. (2017). Impact of the ion transportome of chloroplasts on the optimization of photosynthesis. J. Exp. Bot..

[22] Price C., Williams E., Elhalel G., Sentman D. (2020). Natural ELF fields in the atmosphere and in living organisms. Int. J. Biometeorol..

[23] Price C., Melnikov A. (2004). Diurnal, seasonal and inter-annual variations in the Schumann resonance parameters. J. Atmos. Sol. Terr. Phys..

[24] Price C. (2016). ELF Electromagnetic waves from lightning: The Schumann resonances. Atmosphere.

[25] Elhalel G., Price C., Fixler D., Shainberg A. (2019). Cardioprotection from stress conditions by weak magnetic fields in the Schumann Resonance band. Sci. Rep..

[26] Sakurai T., Yoshimoto M., Koyama S., Miyakoshi J. (2008). Exposure a extremely low frequency magnetic fields affects insulin-secreting cells. Bioelectromagnetics.

[27] Maffei M.E. (2014). Magnetic field effects on plant growth, development, and evolution. Front. Plant Sci..

[28] Da Silva J.A.T., Dobránszki J. (2016). Magnetic fields: How is plant growth and development impacted?. Protoplasma.

[29] Radhakrishnan R. (2019). Magnetic field regulates plant functions, growth and enhances tolerance against environmental stresses. Physiol. Mol. Biol. Plants.

[30] Sarraf M., Kataria S., Taimourya H., Santos L.O., Menegatti R.D., Jain M., Ihtisham M., Liu S. (2020). Magnetic field (MF) applications in plants: An overview. Plants.

[31] Davies M.S. (1996). Effects of 60 Hz electromagnetic fields on early growth in three plant species and a replication of previous results. Bioelectromagnetics.

[32] Aksyonov S.J., Bulychev A.A., Grunina T.Y., Goryachev S.N., Turovetsky V.B. (2001). Effects of ELF-EMF treatment on wheat seeds at different stages of germination and possible mechanism of their origin. Electromagn. Biol. Med..

[33] Iqbal M., Haq Z.U., Jamil Y., Ahmad M.R. (2012). Effect of presowing magnetic treatment on properties of pea. Int. Agrophys..

[34] Fischer G., Tausz M., Köck M., Grill D. (2004). Effects of weak 16 3/2 Hz magnetic fields on growth parameters of young sunflower and wheat seedlings. Bioelectromagnetics.

[35] Rochalska M. (2005). Influence of frequent magnetic field on chlorophyll content in leaves of sugar beet plants. Nukleonika.

[36] Aleman E.I., Mbogholi A., Boix Y.F., Gonzalez-Ohnedo J., Chalfun A. (2014). Effects of EMFs on some biological parameters in coffee plants (*Coffea arabica* L.) obtained by in vitro propagation. Polish, J. Environ. Stud..

[37] Kornarzyński K., Dziwulska-Hunek A., Kornarzyńska-Gregorowicz A., Sujak A. (2018). Effect of electromagnetic stimulation of amaranth seeds of different initial moisture on the germination parameters and photosynthetic pigments content. Sci. Rep..

[38] De Souza-Torres A., Sueiro-Pelegrín L., Zambrano-Reyes M., Macías-Socarras I., González-Posada M., García-Fernández D. (2020). Extremely low frequency non-uniform magnetic fields induce changes in water relations, photosynthesis and tomato plant growth. Int. J. Radiat. Biol..

[39] Yano A., Ohashi Y., Hirasaki T., Fujiwara K. (2004). Effects of a 60 Hz magnetic field on photosynthetic CO_2_ uptake and early growth of radish seedlings. Bioelectromagnetics.

[40] Maxwell K., Johnson G.N. (2000). Chlorophyll fluorescence—A practical guide. J. Exp. Bot..

[41] Porcar-Castell A., Tyystjärvi E., Atherton J., van der Tol C., Flexas J., Pfündel E.E., Moreno J., Frankenberg C., Berry J.A. (2014). Linking chlorophyll a fluorescence to photosynthesis for remote sensing applications: Mechanisms and challenges. J. Exp. Bot..

[42] Kalaji H.M., Schansker G., Brestic M., Bussotti F., Calatayud A., Ferroni L., Goltsev V., Guidi L., Jajoo A., Li P. (2017). Frequently asked questions about chlorophyll fluorescence, the sequel. Photosynth. Res..

[43] Sukhova E., Khlopkov A., Vodeneev V., Sukhov V. (2020). Simulation of a nonphotochemical quenching in plant leaf under different light intensities. Biochim. Biophys. Acta Bioenerg..

[44] Sukhova E., Sukhov V. (2019). Analysis of light-induced changes in the photochemical reflectance index (PRI) in leaves of pea, wheat, and pumpkin using pulses of green-yellow measuring light. Remote Sens..

[45] Murata N., Takahashi S., Nishiyama Y., Allakhverdiev S.I. (2007). Photoinhibition of photosystem II under environmental stress. Biochim. Biophys. Acta Bioenerg..

[46] Ruban A.V. (2015). Evolution under the sun: Optimizing light harvesting in photosynthesis. J. Exp. Bot..

[47] Li X.P., Björkman O., Shih C., Grossman A.R., Rosenquist M., Jansson S., Niyogi K.K. (2000). A pigment-binding protein essential for regulation of photosynthetic light harvesting. Nature.

[48] Li X.P., Gilmore A.M., Caffarri S., Bassi R., Golan T., Kramer D., Niyogi K.K. (2004). Regulation of photosynthetic light harvesting involves intrathylakoid lumen pH sensing by the PsbS protein. J. Biol. Chem..

[49] Demmig-Adams B., Adams III W.W. (1996). The role of xanthophyll cycle carotenoids in the protection of photosynthesis. Trends Plant Sci..

[50] Müller P., Li X.P., Niyogi K.K. (2001). Non-photochemical quenching. A response to excess light energy. Plant Physiol..

[51] Jajoo A., Mekala N.R., Tongra T., Tiwari A., Grieco M., Tikkanen M., Aro E.M. (2014). Low pH-induced regulation of excitation energy between the two photosystems. FEBS Lett..

[52] Singh-Rawal P., Jajoo A., Mathur S., Mehta P., Bharti S. (2010). Evidence that pH can drive state transitions in isolated thylakoid membranes from spinach. Photochem. Photobiol. Sci..

[53] Sukhov V. (2016). Electrical signals as mechanism of photosynthesis regulation in plants. Photosynth. Res..

[54] Flügge U.I., Freisl M., Heldt H.W. (1980). The mechanism of the control of carbon fixation by the pH in the chloroplast stroma: Studies with acid mediated proton transfer across the envelope. Planta.

[55] Wolosiuk R.A., Ballicora M.A., Hagelin K. (1993). The reductive pentose phosphate cycle for photosynthetic CO2 assimilation: Enzyme modulation. FASEB J..

[56] Shikanai T., Yamamoto H. (2017). Contribution of cyclic and pseudo-cyclic electron transport to the formation of proton motive force in chloroplasts. Mol. Plant..

[57] Ettinger W.F., Clear A.M., Fanning K.J., Peck M.L. (1999). Identification of a Ca^2+/^H^+^ antiport in the plant chloroplast thylakoid membrane. Plant Physiol..

[58] Stange B.C., Rowland R.E., Rapley B.I., Podd J.V. (2002). ELF magnetic fields increase amino acid uptake into *Vicia faba* L. roots and alter ion movement across the plasma membrane. Bioelectromagnetics.

[59] Pazur A., Rassadina V., Dandler J., Zoller J. (2006). Growth of etiolated barley plants in weak static and 50 Hz electromagnetic fields tuned to calcium ion cyclotron resonance. Biomagn. Res. Technol..

[60] Betti L., Trebbi G., Fregola F., Zurla M., Mesirca P., Brizzi M., Borghini F. (2011). Weak static and extremely low frequency magnetic fields affect in vitro pollen germination. Sci. World J..

[61] Belyavskaya N.A. (2004). Biological effects due to weak magnetic field on plants. Adv. Space Res..

[62] Goldsworthy A., Volkov A.G. (2006). Effects of electrical and electromagnetic fields on plants and related topics. Plant Electrophysiology. Theory and Methods.

[63] Pazur A., Rassadina V. (2009). Transient effect of weak electromagnetic fields on calcium ion concentration in Arabidopsis thaliana. BMC Plant Biol..

[64] Krupenina N.A., Bulychev A.A. (2007). Action potential in a plant cell lowers the light requirement for non-photochemical energy-dependent quenching of chlorophyll fluorescence. Biochim. Biophys. Acta.

[65] Sukhov V., Surova L., Morozova E., Sherstneva O., Vodeneev V. (2016). Changes in H^+^-ATP synthase activity, proton electrochemical gradient, and pH in pea chloroplast can be connected with variation potential. Front Plant Sci..

