# Supplementary material for: Influence of Magnetic Field with Schumann Resonance Frequencies on Photosynthetic Light Reactions in Wheat and Pea
Source: Cells. 2021 Jan 13;10(1):149. doi: 10.3390/cells10010149 (PMC7828558; doi:10.3390/cells10010149)
Supplement: Supplementary file 1 [file cells-10-00149-s001.zip › Fig. S2.pdf]

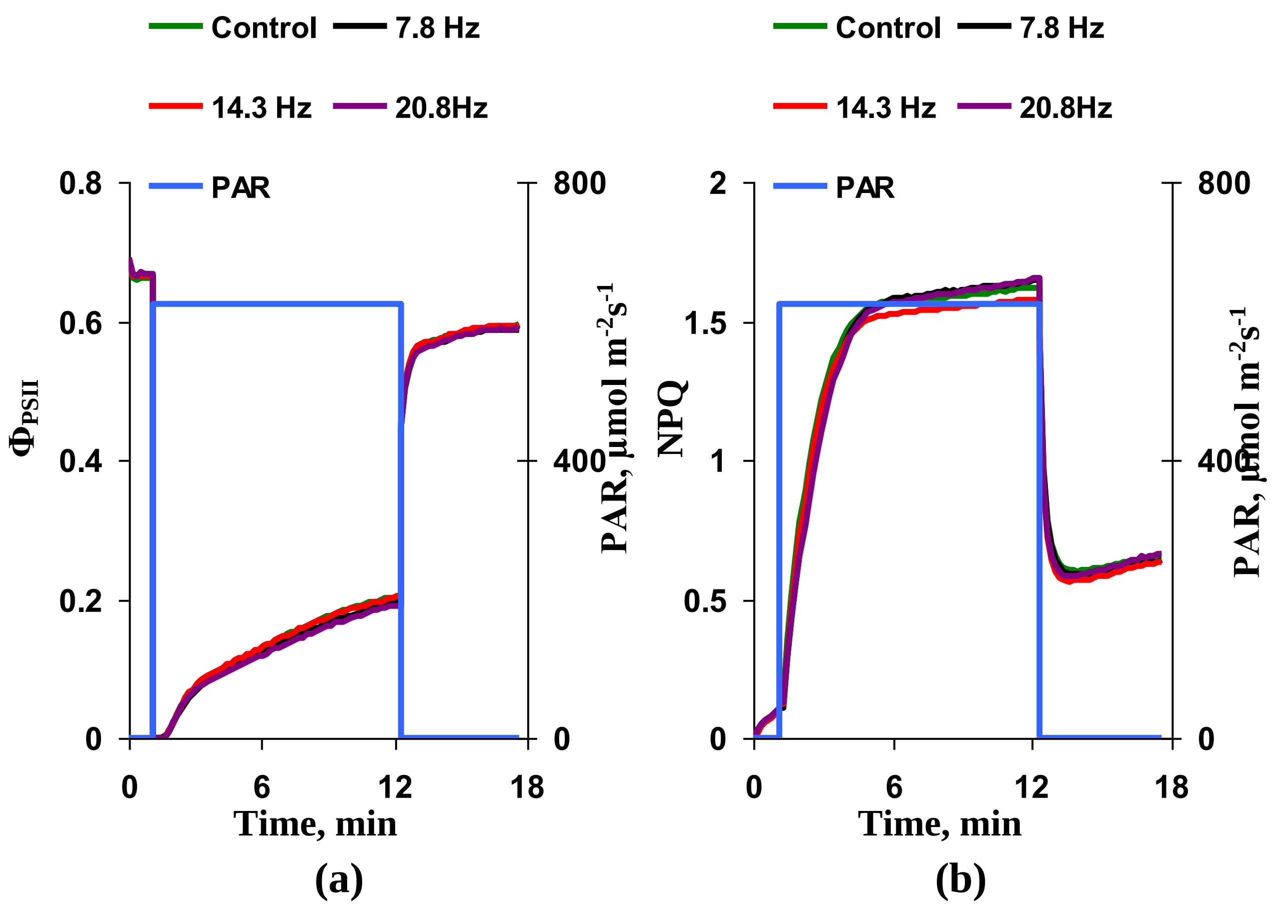

Figure S2. Average dynamics of changes in quantum yield of photosystem II (Φ_{PSII}) **(a)** and non-photochemical quenching (NPQ) **(b)** under action of actinic light (its intensity is marked as PAR) in pea seedlings under short-term action of ELFMFs with different frequencies (*n*=9). Standard errors were not shown. Illumination was initiated after 15 min of dark adaptation. Action of the artificial magnetic field was immediately initiated before initiation of dark adaptation; total duration of its action was 30 min. Photosynthetic parameters were measured under the action of this field. Magnitude of magnetic fields was 18 µT; control plants were not treated by this artificial magnetic field.
