# Supplementary material for: Influence of Magnetic Field with Schumann Resonance Frequencies on Photosynthetic Light Reactions in Wheat and Pea
Source: Cells. 2021 Jan 13;10(1):149. doi: 10.3390/cells10010149 (PMC7828558; doi:10.3390/cells10010149)
Supplement: Supplementary file 1 [file cells-10-00149-s001.zip › Fig. S3.pdf]

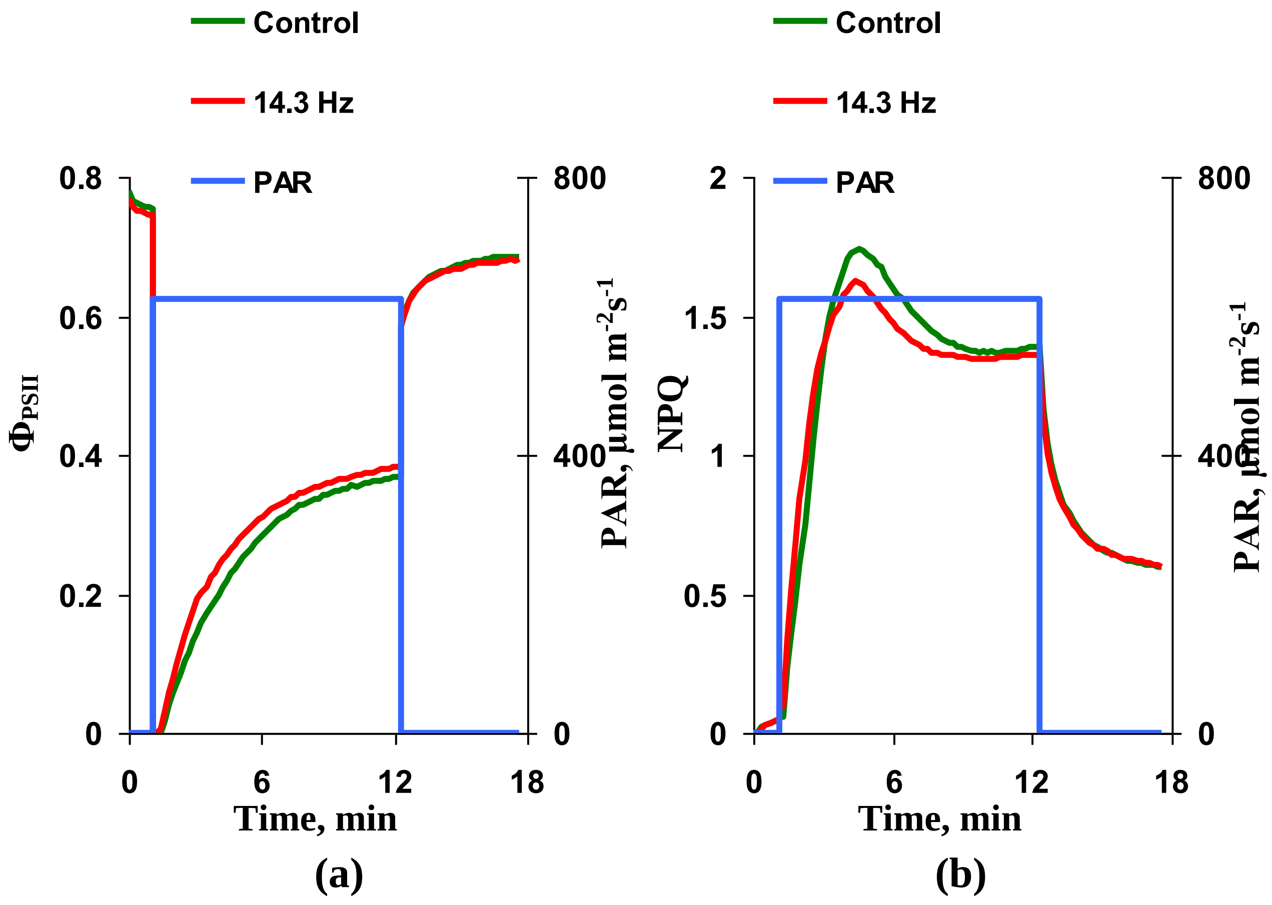

Figure S3. Average dynamics of changes in quantum yield of photosystem II (Φ_{PSII}) **(a)** and non-photochemical quenching (NPQ) **(b)** under action of actinic light (its intensity is marked as PAR) in wheat seedlings under chronic action of ELFMF (*n*=30). Standard errors were not shown. Illumination was initiated after 15 min of dark adaptation. Plants were cultivated under extremely low frequency magnetic field action. Photosynthetic parameters were measured under the action of ELFMF. Magnitude of magnetic fields was 18 µT; frequency was 14.3 Hz. Control plants were not treated by this artificial magnetic field.
